# RESPIRATORY HEALTH: Measuring the Health Effects of Crop Burning

**DOI:** 10.1289/ehp.118-a475

**Published:** 2010-11

**Authors:** Tina Adler

**Affiliations:** **Tina Adler** first wrote for *EHP* about the Clinton–Gore environmental agenda in 1993. She is a member of the National Association of Science Writers and the American Society of Journalists and Authors

What to do with crop residue left in fields at the end of a growing season is, literally, a burning issue. Some farmers prefer the inexpensive approach of setting the stubble ablaze, but repeated burning is not good for the soil,^1^ and the resulting smoke is a health hazard.^2^ Although many studies have measured the particles released into the air by crop burning, fewer have isolated the effect of the smoke on lung function. New research now shows the smoke produced by crop burning could have a lasting effect on children’s lung function.^3^

Ravinder Agarwal, head of the University Science Instrumentation Centre at Thapar University in Patiala, India, and colleagues used portable spirometers to regularly test the lung function of children aged 10–13 and adults aged 20–35 over the course of a year. The 40 participants were healthy nonsmokers living in a village surrounded by farmland, with little traffic and no industry within 10 km.^3^

Children’s force vital capacity (FVC)^4^ dropped from a mean 98% in August 2008 to 92% in July 2009. Mean FVC dipped as low as 88% in October and November, when farmers burned their rice crop residue, and in April and May, when they burned wheat stubble. The children’s mean lung function remained significantly lower throughout the test period. The mean lung function of the adult study participants declined during the burn seasons as well, but largely returned to original levels by the end of the study.^3^

Decreases in lung function correlated with increases in the concentration of particulate matter, which exceeded India’s national air quality standards during the burn season.^3^ Small particles (PM_2.5_ and PM_10_)—which make up the majority of the smoke produced by crop burning—were more closely associated with decreases in lung function than suspended particulate matter (SPM), which can contain particles 100 μm or larger.^5^

The findings linking seasonal burning with health issues “coincide with the anecdotal evidence that we have been seeing in the Canadian prairies,” notes Kate Letkemann, environmental issues coordinator of The Lung Association, Manitoba, and a member of the provincial Crop Residue Burning Advisory Committee. On top of regulations regarding what time of day and where crop residue can be burned,^6^ Manitoba uses incentives to encourage farmers to adopt alternative residue management practices, says Andrew Nadler, coordinator of the governmental Manitoba Crop Residue Burning Program. In the United States, crop burning is regulated at the state level.^7^

Argawal’s work “builds a relationship between pulmonary function tests and the concentration of SPM, PM_10_, and PM_2.5_,” notes Shijian Yang of the School of Environmental Science and Engineering at China’s Shanghai Jiao Tong University. But he would like to see further research that looks closely at the dose–effect relationship between lung function and crop residue burning. Yang’s work has shown that the peak concentration of PM_10_ and its duration may be more important than average concentrations for estimating the health effects of burning crops.^8^
